# Evaluation of pesticide residues in human blood samples from Punjab (India)

**DOI:** 10.14202/vetworld.2015.66-71

**Published:** 2015-01-21

**Authors:** Jasbir Singh Bedi, J. P. S. Gill, P. Kaur, A. Sharma, R. S. Aulakh

**Affiliations:** Department of Veterinary Public Health and Epidemiology, School of Public Health and Zoonoses, Guru Angad Dev Veterinary and Animal Sciences University, Ludhiana, Punjab, India

**Keywords:** dichlorordiphenyl trichloroethan, endosulfan, residues, human blood, Punjab

## Abstract

**Aim:**

The present study was undertaken to estimate the current status of residues of organochlorine pesticides (OCPs), organophosphates (OPs) and synthetic pyrethroids (SPs) pesticides in human blood.

**Materials and Methods:**

Human blood samples were analyzed by gas chromatography and confirmed by gas chromatography-mass spectrometry in selective ion monitoring mode.

**Results:**

The gas chromatographic analysis of human blood samples collected from Punjab revealed the presence of p,p’-dichlorodiphenyl dichloroethylene (DDE), p,p’ dichlorodiphenyl dichloroethane (DDD), o,p’ DDE and β-endosulfan at mean levels of 15.26, 2.71, 5.62 and 4.02 ng/ml, respectively. p,p’ DDE residue was observed in 18.0% blood samples, and it contributes 55% of the total pesticide burden in human blood. The difference of total dichlorordiphenyl trichloroethane (DDT) between different age groups of humans was found to be statistically significant (p<0.05). The difference of DDT and endosulfan between dietary habits, gender and spraying of pesticides was found statistically non-significant, however endosulfan residues were observed only in pesticide sprayer’s population.

**Conclusion:**

Occurrence of p,p’ DDE, p,p’ DDD, o,p’ DDE in human blood indicated restricted use of DDT. However, presence of endosulfan residues in occupationally exposed population is a matter of public health concern.

## Introduction

In India, pesticides are one of the most essential components of modern agricultural technology and have contributed greatly in the increase of agriculture yields and control of vector-borne diseases. Orgnochlorine pesticides (OCPs) are among the most commonly used and favorite pesticides of farming communities in the developing countries like India due to their low cost, versatility against various pests and longer half-life. The environmental conditions in tropical countries are highly conducive to rapid multiplication of pests. Therefore, a wide variety of pesticides is used in tropical countries to combat these crop pests and disease vectors [1]. The detection of OCPs and their degradation products in air, water, soil and sediments, fish, birds and food stuffs from India has become a matter of great concern.

Human beings can be exposed to pesticides either by occupational (manufacturing/formulation of pesticides and during application in the agricultural fields) or non-occupational routes (pollution of the ecosystem through food chain). Exposure to OCPs can cause disruption of the endocrine system, increased risk of breast cancer, endometriosis, hypospadias, cryptorchidias and genotoxic effects [2]. Likewise, exposure to synthetic pyrethroids (SP) causes adverse health effects. Exposure to these pyrethroids is of particular concern during pregnancy as these can easily cross placental barrier and affects fetal development [3]. Furthermore, these have carcinogenic and endocrine disruption potential in mammals [4,5]. India was one of the foremost producer and consumer of OCPs particularly dichlorordiphenyl trichloroethane (DDT) and hexachlorocyclohexane (HCH), till the ban/restriction on their use in late 1990s. Still, a substantial amount of these chemicals are being permitted for malaria control and eradication programs as well as in agriculture [6]. Punjab is among the highest user of pesticides in India [7]. Human blood is the most accessible body fluid for ascertaining the pesticide residue levels. The determination of serum levels of pesticides can be used as a biomarker of exposure for evaluating the health effects at certain levels [8,9]. The magnitude of local environmental pollution is reflected by the determination of the levels in human tissues like blood or adipose [10].

Thus, the present study was planned to estimate current status of residues of OCPs, organophosphates (OPs) and SPs pesticides in human blood as OPs and SPs are replacing the OCPs application in agriculture and vector control.

## Materials and Methods

### Ethical approval

Samples were collected after taking the Approval/Consent from the sample donors.

Regarding the evaluation of the body burden of the pesticide residues, 50 samples of human blood were collected from Bathinda district of Punjab (India), which is cotton growing area and one of the highest users of pesticides. Punjab is an agrarian state with total human population of 27 million [11]. Blood sample of 10 ml per participant was collected and centrifuged at 900 × g. Serum was separated and stored at −20°C until chemical analysis. Samples were collected after taking the approval/consent from the sample donors. Information on age, sex, dietary habits (vegetarian/non-vegetarian), height, weight and history of any health disorders, including any type of cancer was registered on the designed proforma. Information on the use of pesticides for pest control in fields was also obtained.

### Extraction and cleanup of pesticide residues

The high purity standards of OCPs, SPs and OPs pesticides were obtained from Sigma-Aldrich, USA and were used for calibration, recovery tests and quantification of residues in samples. All the organic solvents (E. Merck, India Ltd.) used in the present study were glass distilled before usage. Florisil and anhydrous sodium sulfate (HiMedia, India) was activated at 600°C for 2 h prior to use. Pesticide residues from serum samples were extracted by method of [12], with slight modifications. Solvents used for extraction of pesticide residues were procured from E. Merck, India Ltd. Aliquot of 2.0 ml of serum was equilibrated to room temperature and 1.0 ml of methanol was added; the sample was agitated for 1 min, 5 ml n-hexane: diethyl ether (1:1 v/v) mixture was added and again agitated for 2 min. Further, sample was centrifuged for 5 min at 700 × g. The organic phase was collected, and the aqueous phase was extracted twice with 5 ml of n-hexane: diethyl ether (1:1 v/v) mixture. Collected organic phases were combined and evaporated to 1 ml. Clean-up of the sample was done by USEPA method 3620B using florisil as adsorbent in column chromatography and diethyl ether and hexane as elutent, final reconstitution was done with n-hexane: acetone (1:1 v/v) with final volume of 3 ml [13].

### Estimation and confirmation of pesticide residues

In the present study, estimation of pesticide residues was undertaken using Gas chromatograph (GC) equipped with electron capture detector and flame thermionic detector (Shimadzu 2010 Plus). The GC oven temperature for electron capture detector was programmed for an initial temperature of 170°C withhold time of 13 min, and then increased to 270°C at a rate of 3°C/min with hold time of 20 min. Whereas for flame thermionic detector oven temperature was programmed for an initial temperature of 180°C withhold time for 2 min, then increased to 270°C at a rate of 10°C/min with hold time of 3 min and finally to 280°C at a rate of 5°C/min with hold time of 5 min. The injection port temperature was kept at 280°C and the detectors temperature at 310°C. The concentrations of target pesticide residues in blood samples were quantified by comparing the peak area and retention time of the particular compound in sample extracts to that of the corresponding external standard of pesticide run under the same operating conditions separately. Retention times of pesticide standards are depicted in Table-1. The trueness of the method used was estimated by calculating the recovery from spiked samples with known concentrations. The mean recovery values were ranged from 85.4% to 95.5%. The calculated concentrations of residues in samples were not corrected for recovery. The limit of detection was established as 1 ng/g for OCPs and SPs and 2 ng/g for OPs. The confirmation of pesticide residues detected by GC was done on Gas chromatography-Mass spectrometry (Shimadzu GCMS QP 2010 plus). The mass spectrometer was operated in electron impact mode. The emission current for the ionization filament was set at 80 μA generating electrons with energy of 70 eV. Helium (99.99%) at a flow rate of 0.94 ml/min was used as carrier and collision gas. In the present study, selective ion monitoring (SIM) mode in GCMS for OCPs, SPs and OPs was used considering retention time windows and base peak ion.

**Table-1:** Retention time of pesticides on gas chromatography

Pesticides	Retention time (min)
OCPs and SPs	
α-HCH	13.17
β-HCH	14.93
γ-HCH	15.40
δ-HCH	17.01
Heptachlor	20.34
Fenitrothion	21.78
Aldrin	22.78
Fipronil	26.19
Butachlor	28.32
Dieldrin	29.80
p, p’ DDE	29.89
o, p’ DDE	30.39
Endrin	31.38
β-Endosulfan	32.01
p, p’ DDD	32.62
o, p’ DDT	32.84
Endosulfan sulfate	34.86
p, p’ DDT	35.09
Cyhalothrin (cis, trans)	42.5, 42.83
Permethrin	44.52
Cyfluthrin (four isomers)	46.94, 47.49, 47.85, 48.02
Cypermethrin (four isomers)	48.50, 48.97, 49.37, 49.54
Fenvalerate (two isomers)	53.47, 54.71
Deltamethrin (two isomers)	56.61, 57.75
OPs	
Monocrotofos	6.067
Dimethoate	6.717
Parathion methyl	8.384
Malathion	8.837
Chlorpyrifos	9.215
Fenamiphos	10.973
Profenophos	11.209
Ethion	12.217
Triazophos	13.880
Phosalone	16.307

### Statistical analysis

Data were analysed statistically by using SPSS Microsoft version 11.0.1 for windows (SPSS Inc., IBM, Chicago, Illinois). The correlation of pesticide residues with variables was analyzed by the Karl Pearson correlation coefficient. A p<0.05 was considered as statistically significant.

## Results and Discussion

The mean age of participants was 36.3 years with a range of 18-65 years. The mean body mass index (BMI) was 25.12 kg/m. Of total participants, 6 were women. Fourteen participants reported history of spraying pesticides in their fields without using any protective measures. Among all participants, 13 were addicted to tobacco chewing even during working hours. Nineteen individuals were noticed of having one or more health ailments likely joint pains, generalized body aches, numbness, throat infections, and liver carcinoma

The analysis of serum samples revealed the presence of p,p’ DDE, p,p’ DDD, o,p’ DDE and endosulfan residues. The residues of other OCPs, SPs and OPs were not detected in any of the blood sample. The relative corresponding residue levels of pesticide detected in blood samples (Table-2) indicated that p,p’ DDE was the main metabolite of DDT detected in the present study, followed by o,p’ DDE, β- endosulfan and p,p’ DDD. Residue levels of p,p’ DDE were detected in ten individuals with overall mean level of 15.26 ng/ml and a range of ND-213.6 ng/ml. p,p’ DDE was the also major contaminant detected in human breast milk samples in our previous study [6].

**Table-2:** Concentrations (ng/ml) of pesticide residues in human blood samples

Pesticide	Mean	SD	Range	% Positive^a^	% Proportion^b^
p, p’ DDD	2.71	9.34	ND-39.4	8	10
p, p’ DDE	15.26	40.03	ND-213.6	20	55
o, p’ DDE	5.63	19.50	ND-114.7	10	20
∑ DDT	23.60	42.18	ND-213.6	38	85
β-endosulfan	4.08	11.31	ND-38.9	12	15

SD=Standard deviation, ND=Not detected,

a Samples found to be positive,

b Relative proportion in total residues detected, DDD=Dichlorodiphenyl dichloroethane, DDE=Dichlorodiphenyl dichloroethylene, DDT=Dichlorordiphenyl trichloroethane

The presence of p,p’ DDE and o,p’ DDE may indicate the long-term persistence of DDE metabolites in the human body. Further, metabolism of DDT in the human body leads to conversion into DDE and DDD metabolites. Though DDT use is banned in India, but due to lack of suitable alternative for malaria control, India has been permitted to use up to 10,000 tons of DDT per year for its vector control programs [14]. The absence of active and fresh use metabolites of DDT (p,p’ DDT and o,p’ DDT) thus indicated very limited or no use of DDT in public health programs in the region of the present study. However, in North-East India, Mishra *et al*. [15] reported predominance of p,p’ DDT metabolites in human blood samples in Assam (India) with facts that the region is highly receptive to malaria transmission due to excessive rainfall, high humidity and warmer climates mostly throughout the year. The temporal and spatial comparison of present study with previous studies from India indicated that total DDT levels detected in current study (23.7 ng/ml) were several times lower than the corresponding levels of 7170, 271, 950 and 743 ng/ml reported from different parts of India [16-18]. Furthermore, residues of DDT were observed lower than those recorded in Romania (2420 ng/ml), Spain (4895.8 ng/ml) and Sweden (836.1 ng/ml) [19-21].

In the present study, residues of β-endosulfan were detected in human blood samples at mean level of 4.08 ng/ml. Before, the imposition of the ban, endosulfan was extensively used insecticide in agriculture practices and its widespread usage in India has led to its occurrence in a variety of food items [22]. Thus, the presence of β-endosulfan residues in blood samples in the present study may reflect either environmental exposure during spraying or consumption of food containing excessive levels of this pesticide. Earlier, Pathak *et al*. [23] reported the presence of α- (1.39 ng/ml) and β- endosulfan (0.88 ng/ml) in maternal blood samples in India, while Torres *et al*. [24] reported only β- endosulfan residues at level of 76.38 ng/ml in pregnant women blood samples from Spain.

### Factors associated with the occurrence of pesticide residues in human

**Figure-1:**
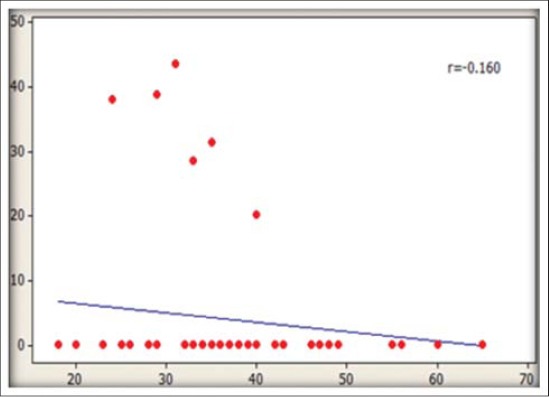
Association of dichlorordiphenyl trichloroethane residues with age of sample donors

**Figure-2:**
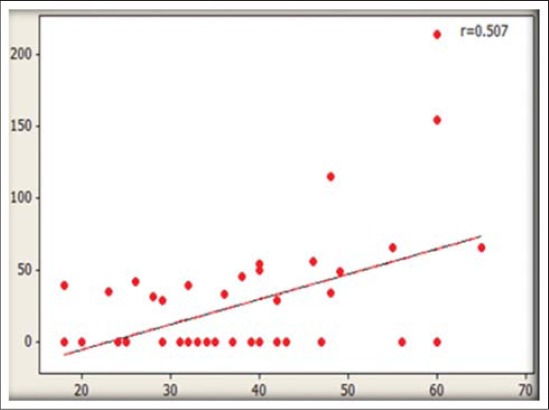
Association of endosulfan residues with age of sample donors

The residue levels of DDT and β-endosulfan in association with various factors (Table-3) indicated that highest mean concentration of total DDT was observed in the age group of >51 years with 66.6% of detection frequency. A strong and positive correlation of DDT residues with age of participants was noticed (r=0.507) while for endosulfan negative correlation (r=−0.160) was observed with age (Figure-1-2). Mann–Whitney U-test results indicated statistically significant differences for total DDT values in all the age groups (p<0.05). Increased residue levels of DDT with the age of human were also reported earlier [20]. Though, statistically non-significant (p>0.05) higher levels of total DDT residues were detected in non-vegetarians (27.21 ng/ml) than vegetarians (16.61 ng/ml). DDT is lipophilic and persistent in nature, accumulates in the fat-rich tissues of animal food and do not get metabolized or excreted. Higher quantities of DDT in non-vegetarians have also been reported earlier [18]. Statistically non-significant (p>0.05) difference between male and female population was observed for total DDT and β-endosulfan residues. However, higher prevalence of DDT concentration in females has also been observed in few other findings [25-26].

**Table-3:** Mean residue levels and standard deviation (ng/ml) of DDT metabolites and β-endosulfan in human blood in relation to different factors

Age groups (years)	p, p’ DDD	p, p’ DDE	o, p’ DDE	∑DDT	β-endosulfan
<20-30 (n=16)	4.24±11.76 (2)	1.96±7.87 (1)	4.84±13.29 (2)	11.05±17.18 (5)	4.82±13.17 (3)
31-40 (n=17)	1.97±8.12 (1)	5.89±16.70 (2)	5.24±14.91 (2)	13.11±21.34 (5)	6.87±13.85 (3)
41-50 (n=11)	3.10±10.28 (1)	12.14±21.68 (3)	10.42±34.58 (1)	25.67±36.60 (5)	ND
51->60 (n=6)	-	83.03±85.40 (4)	-	83.03±85.40 (4)	ND
Dietary habits					
Non-vegetarian (n=32)	2.29±9.07 (2)	19.31±47.73 (7)	4.82±21.24 (2)	26.43±50.05 (11)	2.74±8.98 (3)
Vegetarian (n=18)	1.77±5.22 (2)	6.96±18.56 (3)	5.10±14.89 (3)	16.61±22.76 (8)	6.28±14.62 (3)
Gender					
Male (n=44)	3.08±29.51 (4)	11.77±29.51 (8)	5.43±9.92 (4)	20.28±33.59 (16)	4.56±19.95 (6)
Female (n=6)	-	40.84±85.65 (2)	7.05±17.26 (1)	47.89±83.23 (3)	-
Spraying					
Pesticide sprayer (n=14)	2.39±8.95 (1)	13.22±22.47 (4)	6.05±15.63 (2)	21.66±23.66 (7)	14.35±17.98 (6)
Pesticide non-sprayer (n=36)	2.832±9.619 (3)	16.057±45.316 (6)	5.463±21.007 (3)	24.463±47.765 (12)	
Tobacco chewing/smoking (sprayers)					
Tobacco chewer (n=5)	6.69±14.97 (1)	10.92±24.41 (1)	7.04±15.74 (1)	24.66±23.99 (3)	10.32±14.67 (2)
Non-tobacco chewer (n=9)	3.78±11.36 (1)	3.24±9.73 (1)	-	7.03±14.01 (2)	7.50±15.10 (2)

Figures in parenthesis are indicating the number of positive samples, n=number of samples, ND=Not detected, DDD=Dichlorodiphenyl dichloroethane, DDE=Dichlorodiphenyl dichloroethylene, DDT=Dichlorordiphenyl trichloroethane

**Figure-3:**
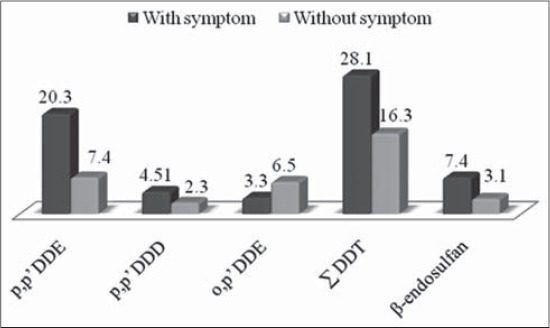
Comparative dichlorordiphenyl trichloroethane and endosulfan residue levels in relation to any clinical symptom

Residues of endosulfan were noticed only in blood samples of individuals who were regularly involved in pesticides spraying operations without using any protective measures. Among pesticides sprayers, five were tobacco chewers/smokers and it was observed that residues of β-endosulfan were observed more in them when compared with non-tobacco chewers/smokers (Table-3). Dirtu *et al*. [20] have observed comparatively more residue levels of total OCPs in blood of tobacco-addicted population (4326 ng/ml) than non-addicted (3856 ng/ml). However, in this study residue levels of only p,p’ DDD were observed higher in tobacco chewer (4.76 ng/ml) than non-tobacco chewer (1.98 ng/ml). In addition, we also observed that pesticide sprayers spray pesticides with naked hands and usually chew tobacco and smoke in between spraying operation, which may also lead to intake of pesticides from their contaminated hands. In the present study, it was noticed that participants with either one or more symptoms (joint pain, numbness, depression, headache and throat infections) have more residue levels of total DDT and β-endosulfan in comparison to participants without any illness (Figure-3). Khan *et al*. [27] also reported headache, dizziness, vomiting, shortness of breath, skin rash, fatigue and tiredness among pesticides exposed workers. In our study, the blood sample of a participant (46-year-old) suffering from liver cancer was found to contain p,p’ DDE metabolite at level of 55.7 ng/ml. Though long-term exposure to DDT, DDE, or DDD induced liver cancer in mice [28] but in human, this fact can be validated by increasing the sample size, analysis of biochemical profile and recording more epidemiological features in different populations.

## Conclusions

The present study did an analysis of a wide variety of pesticides in human blood samples from the cotton belt of Punjab. Occurrence of p,p’ DDE, p,p’ DDD, o,p’ DDE in human blood indicated restricted use of DDT. However, presence of endosulfan residues in both non-occupational and occupational exposed population is a matter of public health concern. The results showed that older age, pesticide spraying activities and non-vegetarian dietary habits are associated with higher levels of pesticide burden. As the health consequences of pesticides, residue levels in human blood are uncertain. Therefore future monitoring studies are required to assess the relation between residue levels and their deleterious effects particularly cancer on human health and environment.

## Authors Contributions

JSB, PK and AS collected the human blood samples, did their analysis including extraction and clean up of pesticide residues from sample matrix. They also did data analysis. JPSG and RSA helped in standardization of multiresidue method and data analysis. All authors participated in draft and revision of the manuscript. All authors read and approved the final manuscript.
